# Automated online safety margin (GLIOVIS) for glioma surgery model

**DOI:** 10.3389/fonc.2024.1361022

**Published:** 2024-04-29

**Authors:** Marianne Mazevet, Christian Oberli, Sebastiano Marinelli, Ismail Zaed, Stefanie Bauer, Alain Kaelin-Lang, Francesco Marchi, Roberto Gardenghi, Michael Reinert, Andrea Cardia

**Affiliations:** ^1^ Department of Innovative Technologies, University of Applied Sciences and Arts of Southern Switzerland, Lugano, Switzerland; ^2^ Department of Neurosurgery, Neurocenter of Southern Switzerland, Ente Ospedaliero Cantonale, Lugano, Switzerland; ^3^ Faculty of Biomedical Sciences, Università della Svizzera Italiana, Lugano, Switzerland; ^4^ Department of Neurology, Neurocenter of Southern Switzerland, Ente Ospedaliero Cantonale, Lugano, Switzerland; ^5^ Department of Neurology, Inselspital, Bern University Hospital, University of Bern, Bern, Switzerland; ^6^ Department of Neurosurgery, Hirslanden Neurological and Spinal Surgery Center, St. Anna Clinic, Lucerne, Switzerland; ^7^ Department of Neurosurgery, Inselspital University Hospital, University of Bern, Bern, Switzerland

**Keywords:** glioblastoma, 5-ALA–guided surgery, fluorescence quantification, safety margins, gliolan

## Abstract

**Purpose:**

Glioblastoma is the most common type of primary brain malignancy and has a poor prognosis. The standard treatment strategy is based on maximal safe surgical resection followed by radiotherapy and chemotherapy. Surgical resection can be optimized by using 5-delta-aminolevulinic acid (5-ALA)–induced fluorescence, which is the current mainstay. Although 5-ALA–induced fluorescence has gained general acceptance, it is also limited by inter-observer variability and non-standardized fluorescence parameters. We present a new software for processing images analysis to better recognize the tumor infiltration margins using an intraoperative immediate safety map of 5-ALA–induced fluorescence. We tested this in a brain model using a commercial surgical exoscope.

**Methods:**

A dedicated software GLIOVIS (ACQuF-II, Advanced Colorimetry-based Quantification of Fluorescence) was designed for processing analysis of images taken on the Intraoperative Orbital Camera Olympus Orbeye (IOC) to determine the relative quantification of Protoporphyrin IX (5-ALA metabolite) fluorescence. The software allows to superpose the new fluorescence intensity map and the safety margins over the original images. The software was tested on gel-based brain models.

**Results:**

Two surrogate models were developed: PpIX agarose gel–integrated in gelatin-based brain model at different scales (1:25 and 1:1). The images taken with the IOC were then processed using GLIOVIS. The intensity map and safety margins could be obtained for all available models.

**Conclusions:**

GLIOVIS for 5-ALA–guided surgery image processing was validated on various gelatin-based brain models. Different levels of fluorescence could be qualitatively digitalized using this technique. These results need to be further confirmed and corroborated *in vivo* and validated clinically in order to define a new standard of care for glioblastoma resection.

## 1 Introduction

The clinical outcome of glioblastoma patients is poor with a median overall survival of approximately 15–18 months (1–4). The extent of resection defined as the removal of the contrast-enhancing (CE) tumor is directly correlated with overall survival and progression-free survival (5–8).

Fluorescence-guided surgery has emerged as a sensitive and effective method to define tumor location and delineate its margins during the procedure, maximizing the extent of resection (9–11). Several fluorescent agents have been assessed in clinical trials over the past few years for malignant glioma including 5-aminolevulinic acid (5-ALA), fluorescein, indocyanine green, hypericin, 5-aminofluorescein bound to human serum albumin, and endogenous spectroscopy (9, 12). However, 5-ALA is the only agent that has been tested in a multi-center randomized controlled trial that has been approved by the Food and Drug Administration and by the European Medicines Agency (13, 14). Furthermore, 5-ALA–guided surgery is limited by inter-observer subjectivity and by the variability and the lowering of fluorescence intensity at the tumor margins (15). Moreover, the microscopic visualization of the PpIX fluorescence needs repeated transitions between white light and blue light, resulting in time consuming surgeries and risk of injuries of critical structures (12).

In addition to these limitations, when the normal brain overlaps the pathological tissue or the orientation of the microscope view is not appropriate (“non-orthogonal working corridors” or “dark corridors”), no fluorescence can be seen under the blue light filter (11). Similarly, blood, CSF, cottonoids, or other hemostatic agents can hide fluorescent tissue and limit the extent of resection. Therefore, the surgeon’s eye appears to be not always reliable for the identification of the tumor-brain interface.

Over the past few years, our multidisciplinary team (neurosurgeons, biologists, and engineers) dedicated its efforts to design an intraoperative microscope able to provide an intensity map (IM) of the fluorescence and the tumor safety margins (SMs).

This project started with the construction of our custom-made microscope (Qp9), and several upgrades of the hardware and of the software were implemented, leading to the actual concept. We present the software GLIOVIS (ACQuF-II, Advanced Colorimetry-based Quantification of Fluorescence), which provides both fluorescence IM and well-defined SMs based on relative digital fluorescence quantification of 5-ALA–induced protoporphyrin (PpIX). This new technique may possibly lead and support the surgeon for optimal resection of high-grade glioma (HGG) lesions of the brain.

## 2 Methods

GLIOVIS was developed for post-processing the fluorescent images taken by the Intraoperative Orbital Camera Olympus Orbeye (IOC). The core of the code consists of a specific Colorimetry Camera–Based Algorithm (CCBA) for the relative quantification of different fluorescence intensity levels within the same sample and thus generating the IM allowing a better visualization of the tumor-mimicking samples. The CCBA was applied on original RGB (red, green, and blue) images taken by the IOC with samples illuminated by blue light. Each original image (OI) was post-processed so that a filtered active fluorescence image (AFI) was extracted based on RGB colorimetry approaches. Subsequently, an intensity threshold mask was applied to the AFI in order to obtain a Boolean mask image (BMI). Thereafter, the IM with relative fluorescence colored scale was generated by merging both images AFI and BMI. Finally, the SM was outlined by extracting the contour from the BMI. Both the IM and SM were overlapped with the OI, resulting in an improved visualization of the area corresponding to the tumor mimicking samples.

A proof-of-concept 3D brain phantom with integrated tumor-surrogate mass was developed in two different scales: small-brain model (1:25) and half-brain model (1:1). Both brain models consisted of gelatin and the tumor-surrogate mass was made of agarose gel containing a known concentration of PpIX. The developed brain models represent a known and stable fluorescence source that simulate the tumor fluorescence emission, which in the real tumor is created by cell metabolism starting from 5-ALA.

The purpose of the 3D brain phantom was limited to the preliminary assessment of the sensitivity of the IOC combined with the new post-processing software GLIOVIS. The suitability of the 3D brain phantom in faithfully reproducing the tumor infiltrations was also rather limited.

**Figure 1:**
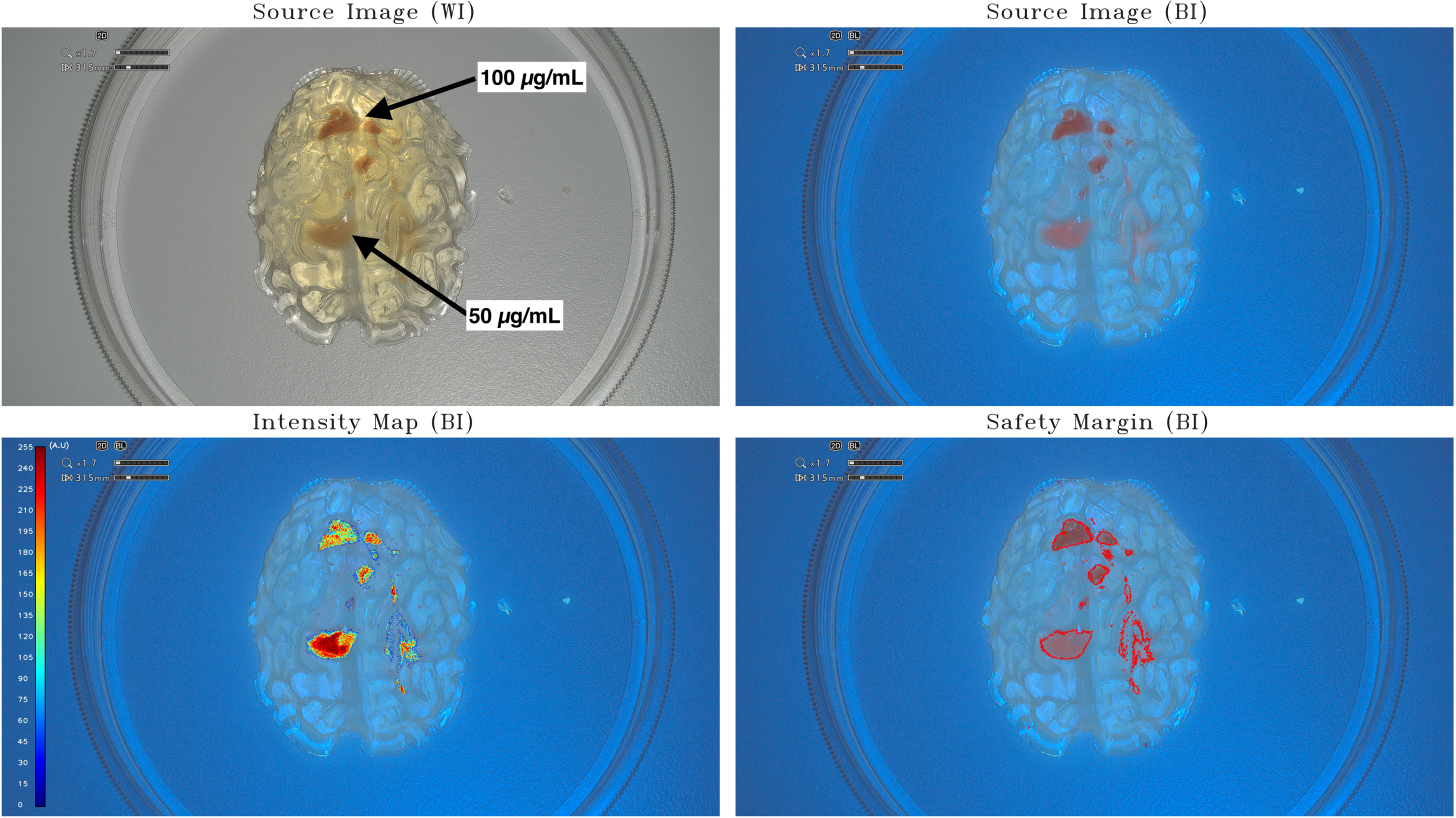
Intensity map and safety margins on small-brain model. On the top: IOC source image of the small-brain model (scale, 1:25) integrating tumor-surrogate masses illuminated with white light (WI; left) and with blue light (BI; right). On the bottom: IOC source image of the small-brain model (scale, 1:25) integrating tumor-surrogate masses illuminated with blue light and overlapped intensity map (left) and safety margins (right). The latter are determined with GLIOVIS. The fluorescent zones with high PpIX concentration (100 µg/mL) are quantified with a mean value of around 190 au, whereas lower PpIX concentrations (50 µg/mL) are quantified with higher mean value of around 250 au. The origin of this phenomenon could be found in the fact that the tumor-surrogate mass with the higher concentration lies deeper in the model and, therefore, the greater attenuation of the light falsifies the result by making a lower quantification appear.

**Figure 2:**
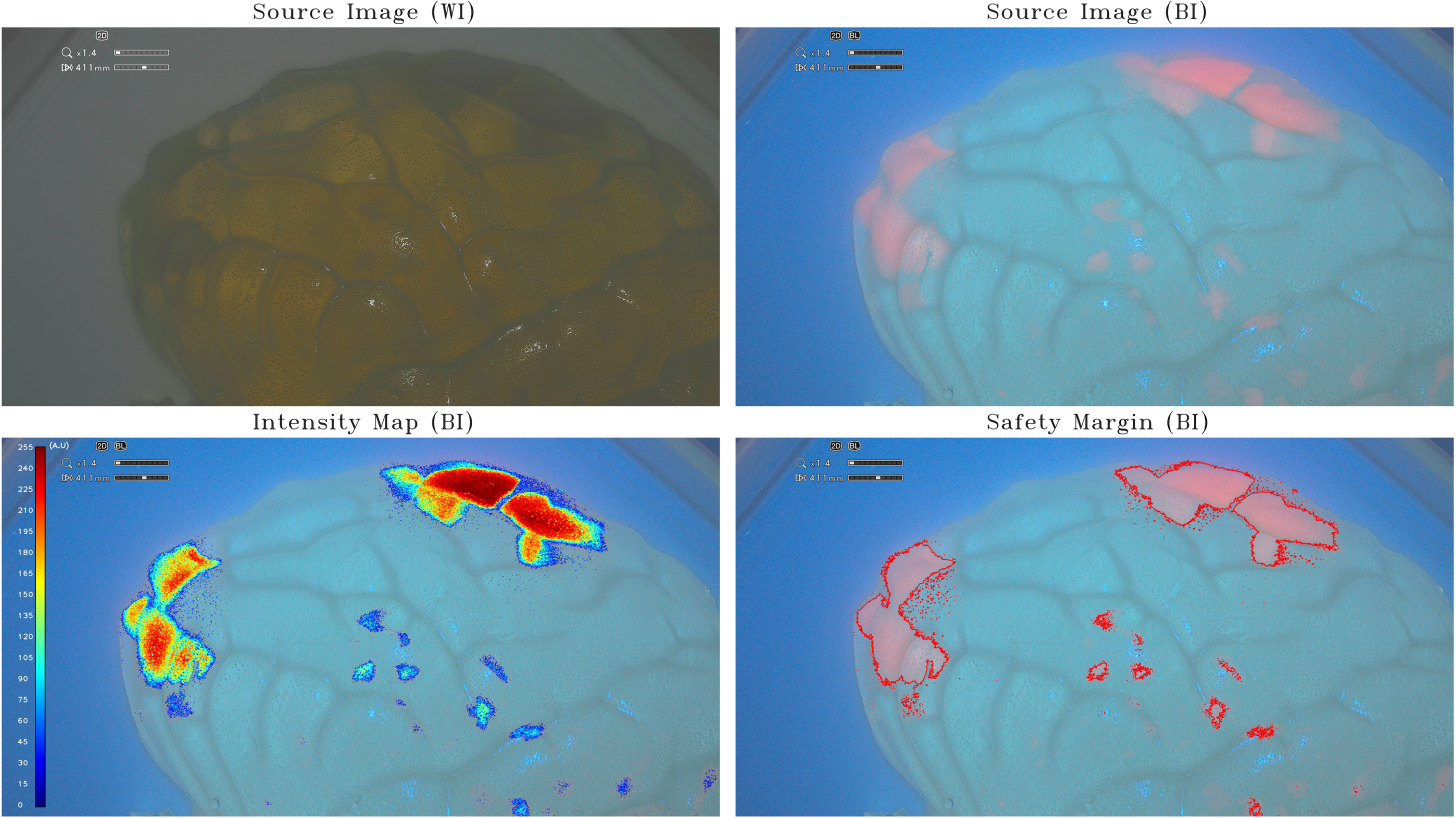
Intensity map and safety margins on half-brain model. On the top: IOC source image of the half-brain model (scale, 1:1) integrating tumor-surrogate masses illuminated with white light (WI; left) and with blue light (BI; right). On the bottom: IOC source image of the half-brain model (scale, 1:1) integrating tumor-surrogate masses illuminated with blue light and overlapped intensity map (left) and safety margins (right). The latter are determined with GLIOVIS. All shown fluorescent zones have the same PpIX concentration (5 µg/mL). As for the small-brain model in **Figure 1**, the quantification of fluorescence depends on the depth of the tumor-surrogate mass. The two areas on the top right have a higher quantified fluorescence (250 au) than those on the left (180 au), despite the concentration being the same (5 µg/mL).

**Figure 3:**
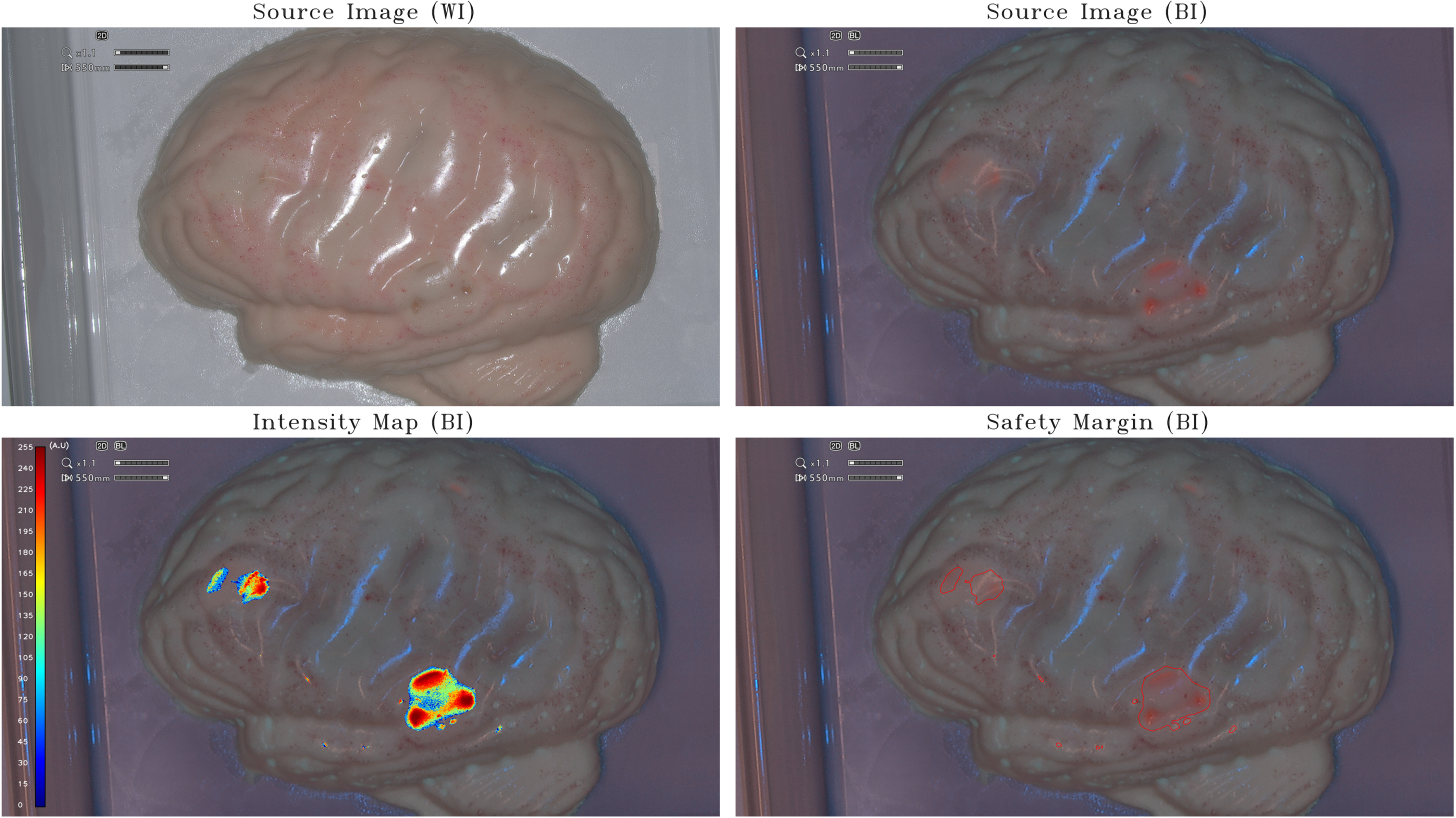
Intensity map and safety margins on optimized half-brain model. On the top: IOC source image of the optimized opaque-pinkish half-brain model (scale, 1:1) integrating tumor-surrogate masses illuminated with white light (WI; left) and with blue light (BI; right). On the bottom: IOC source image of the optimized opaque-pinkish half-brain model (scale, 1:1) integrating tumor-surrogate masses illuminated with blue light and overlapped intensity map (left) and safety margins (right). The latter are determined with GLIOVIS. The shown fluorescent zones have various PpIX concentrations between 1.25 μg/mL and 5 μg/mL. As for the small-brain model (**Figure 1**) and the half-brain model (**Figure 2**), the quantification of fluorescence depends on the depth of the tumor-surrogate mass. Therefore, also in this case, the quantified fluorescence is not direct proportional with the PpIX concentration.

The assessment phase on the available IOC took place over 5 weeks (from 18 April 2023 to 26 May 2023). A first acquisition session was performed using the small-brain model (scale, 1:25) with the incorporation of PpIX agarose gels at a relative high concentration of 50 µg/mL and 100 µg/mL (**Figure 1**). The second acquisition was obtained by inserting in a half-brain model (scale, 1:1) PpIX agarose gels at a lower concentration of 0.5 µg/mL and 5 µg/mL (**Figure 2**). The third step was to acquire the images in an improved half-brain model (scale, 1:1) with modulation of opacity and color (**Figure 3**).

The third acquisition sessions mentioned above were intended as very preliminary tests to verify the range of sensitivity of the IOC and not to exactly quantify the concentration of PpIX. Moreover, because the fluorescent zone was inside the brain model, the attenuation of the light was not uniform but dependent of the thickness of the gelatin layer.

**Figure 4:**
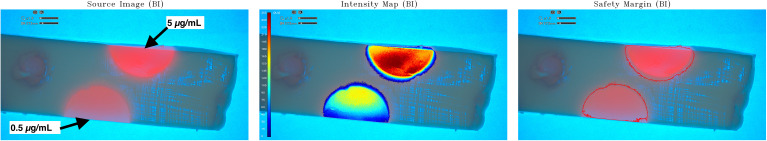
Intensity map and safety margin on optimized half-brain model coronal sections. Coronal sections were made on optimized brain models to observe depth of fluorescence and relative quantification on two tumor-surrogate different PpIX concentrations (0.5 µg/mL and 5 µg/mL). On the left: The source image with blue illumination. The intensity map and safety margins of sections illuminated with blue light were displayed in the middle and right, respectively. Two different levels of fluorescence could be observed in these pictures: at the top, the PpIX gel of 5μg/mL; and at the bottom, the PpIX gel of 0.5μg/mL. The fluorescent spot on the bottom (PpIX concentration of 0.5 µg/mL) shows relative concentrations between 0 au and 150 au (mean value of 120 au), whereas the one on the top (PpIX concentration of 5 µg/mL) shows relative concentrations between 0 au and 255 au (mean value of 240 au). The higher PpIX concentrations show higher relative values than the lower ones. The relative quantified fluorescence of the tumor-surrogate mass in this case is proportional with the PpIX concentration, because the light is not attenuated by the brain model gelatin.

Finally, the last acquisition session was performed on a coronal section of the half-brain model with inside two different tumor-surrogate masses representing two different PpIX gel concentrations (0.5 µg/mL and 5 µg/mL). The depth of fluorescence and the different level of fluorescence intensity were obtained (**Figure 4**).

Although the analysis was done retrospectively, the software may be adapted to any RGB camera system (Exoscope, Microscope) with the adequate interface for immediate imaging.

## 3 Results

The IM was obtained for all 3D brain models used in the experiment (small-brain model, half-brain model, and half-brain model with modulation of opacity and color).

The first raw images obtained with the incorporation of PpIX gels (50 µg/mL and 100 µg/mL) in a gelatin-based model (scale, 1:25) were processed using the software GLIOVIS: the IM and the SM visualization of the tumor mock are shown in **Figure 1**. The IM obtained in this case must be considered a positive preliminary result confirming that PpIX concentrations in the range between 50 µg/mL and 100 µg/mL can be easily detected and relative-quantified by post-processing the images of the IOC by means of the GLIOVIS software. The fluorescent zones with high PpIX concentration (100 µg/mL) are quantified with a mean value of around 190 au, whereas lower PPIX concentrations (50 µg/mL) are quantified with higher mean value of around 250 au. The origin of this phenomenon could be found in the fact that the tumor-surrogate mass with the higher concentration lies deeper in the model and, therefore, the greater attenuation of the light falsifies the result by making a lower quantification appearance.

The same tests were performed on a half-brain model (scale, 1:1) using PpIX agarose gels at a much lower concentrations of 0.5 µg/mL and 5 µg/mL, the latter corresponding to the maximum PpIX concentration within glioblastoma tumors (16). This resulted in a more realistic effect as shown in **Figure 2**. All shown fluorescent zones have the same PpIX concentration (5 µg/mL). As for the small-brain model (**Figure 1**), the quantification of fluorescence depends on the depth of the tumor-surrogate mass. The two areas on the top right have a higher quantified fluorescence (250 au) than those on the left (180 au), despite the concentration being the same (5 µg/mL).

To limit the light reflection on the sample and to be more similar to the human brain, the half-brain model was optimized to render it opaque with a pinkish color (**Figure 3**). The IM obtained in the last two cases proves that also lower PpIX concentrations within the range existing inside real glioblastoma tumors can be detected and relative-quantified on a 1:1 scale 3D brain phantom by post-processing the images of the IOC by means of the GLIOVIS software. As for the small-brain model (**Figure 1**) and the half-brain model (**Figure 2**), the quantification of fluorescence depends on the depth of the tumor-surrogate mass. Consequently, also in this case, the quantified fluorescence is not directly proportional with the PpIX concentration.

The half-brain model considered above (**Figure 3**) was then sectioned (coronal sections to observe the depth of fluorescence and the different levels of fluorescence intensity on two PpIX gels (0.5 µg/mL and 5 µg/mL) as represented in **Figure 4**. Compared to the source image (left), the GLIOVIS software post-processed image (middle) shows more clearly the PpIX concentration level, and the limits of the PpIX presence are clearly recognizable. The relative quantification visible on the IM (image in the middle) must be further investigated, but, considering that the colored scale is not linear and the camera/illumination system was not calibrated, the qualitative preliminary result obtained has to be considered positive. The fluorescent spot on the bottom (PpIX concentration of 0.5 µg/mL) shows relative concentrations between 0 au and 150 au (mean value of 120 au), whereas the one on the top (PpIX concentration of 5 µg/mL) shows relative concentrations between 0 au and 255 au (mean value of 240 au). The higher PpIX concentrations shows higher relative values than the lower ones.

## 4 Discussion

Recently, the “RANO resect group” presented data demonstrating that more important than the extent of resection is the measurement of the remaining tumor tissue (17). Therefore, the removal of non-CE tumors beyond the CE tumor borders is considered to give additional survival benefit in the so-denominated “supramaximal CE resection” (17, 18). 5-ALA is the agent that provides real-time fluorescent guidance to the neurosurgeon in order to perform a more complete resection of HGGs (19, 20). Nevertheless, it is widely known that glioblastoma does not accumulate 5-ALA–induced fluorescence homogenously: heterogeneity has been demonstrated in relation to the tumor grade, the tumor cell density, the cellular proliferation indices, the infiltration, and, particularly, at the border of the MRI enhanced lesion (21, 22). Moreover, the heterogeneous intensity of PpIX fluorescence has also been strongly correlated with the expression of genetic features and biomolecular markers, such as the expression of epithelial growth factor receptor and its downstream effect on Heme oxygenase-1 as already demonstrated by our group in a previous work (23, 24). Therefore, quantification of fluorescence represents the next level of information for surgeons, being useful for the identification of areas with lower levels of PpIX accumulation and thus accurate SM mapping (21). Over the past few years, our multidisciplinary team (neurosurgeons, biologists, and engineers) dedicated its efforts to design an intraoperative microscope able to provide an IM of the fluorescence and of the tumor SMs (24–26). Our custom-made microscope (Qp9), of which the basic functionality was already described previously by Valdes et al., was progressively implemented in order to reach real-time processing for appropriate intraoperative use (27, 28). The software GLIOVIS (ACQuF-II, Advanced Colorimetry-based Quantification of Fluorescence) provides both fluorescence IM and well-defined SMs based on the relative digital fluorescence quantification of 5-ALA–induced protoporphyrin (PpIX). The assessment of the Olympus Orbeye orbital camera system has offered the possibility of validating the specific post-processing software GLIOVIS and the brain models on a commercial high-quality surgical device (**Supplementary Figure 1**). Finally, the developed software GLIOVIS was shown to be able to qualitatively differentiate PpIX concentrations in a 3D Glioma brain phantom in an online fashion and produce a digitalized quantified image (**Figure 4**). This new technique may possibly lead and support the surgeon for optimal resection of HGG lesions of the brain thanks to the better visualization of the SM and, particularly, to the possibility of achieving a semi-quantitative system of real-time visualization of the fluorescent and non-fluorescent tumor field.

### 4.1 Limitations and future perspectives

The software GLIOVIS applied to the single pictures and video stream allowed us to qualitatively elaborate the IM as well as the SMs of all our brain models (**Supplementary Figure 2**).

However, several limitations must be highlighted concerning the present feasibility study.

The post-processing of the images with the software did not consider the variability in intensity fluorescence based on the angulation of the IOC light field while it impacts the surface. Secondarily, further development efforts are needed to retrieve Orbeye images in live stream, by integrating GLIOVIS directly into the exoscope or the microscope system, displaying the SM onto the surgeon’s visual interface (screen, microscope, and goggles). To achieve this goal, it will be necessary to implement both reference camera and illumination systems and to further improve the software GLIOVIS mentioned above, considering external parameters such as the intensity emitted by the light source.

This enhancement will provide the surgeon with high accuracy and repeatability of the fluorescence measurements in different environmental conditions.

This system will be further tested *ex vivo* on animals and on human glioblastoma brain tissue samples. An intraoperative human surgical use will further require a CE certification for intraoperative use in a commercially available surgical exo/microscope.

## 5 Conclusion

Accurate discrimination between tumor borders and normal tissue is crucial to maximize tumor resection, reduce contrast enhanced and non-contrast enhanced residual tumor volume, and, overall, to improve OS and PFS. Qualitative fluorescence of PpIX has been used for this purpose in HGG but its quantification remains an open field of research. Our novel method, which combines the Intraoperative Olympus Orbeye Camera with the software GLIOVIS, proved the preliminary feasibility of creating an IM of PpIX fluorescence and the SMs in all our 3D brain phantoms, at this time, in post-acquisition. Integrating the software GLIOVIS directly into the microscope or exoscope will allow the online depiction of the SM image in less than a second. Further investigations and *ex vivo* analysis are required to proceed to the request for the permission to use the concept in *in vivo* human use.

## Data availability statement

The original contributions presented in the study are included in the article/**Supplementary material**. Further inquiries can be directed to the corresponding author.

## Author contributions

MM: Validation, Visualization, Writing – original draft, Writing – review & editing, Data curation, Formal analysis, Software. CO: Data curation, Formal analysis, Software, Validation, Visualization, Writing – original draft, Writing – review & editing. SM: Data curation, Formal analysis, Software, Validation, Visualization, Writing – original draft, Writing – review & editing. IZ: Validation, Visualization, Writing – original draft, Writing – review & editing. SB: Visualization, Writing – original draft, Writing – review & editing. AK-L: Visualization, Writing – original draft, Writing – review & editing, Supervision, Validation. FM: Validation, Visualization, Writing – original draft, Writing – review & editing. RG: Conceptualization, Funding acquisition, Methodology, Project administration, Supervision, Validation, Visualization, Writing – original draft, Writing – review & editing. MR: Conceptualization, Funding acquisition, Methodology, Project administration, Supervision, Validation, Visualization, Writing – original draft, Writing – review & editing. AC: Validation, Visualization, Writing – original draft, Writing – review & editing.

## Supplementary material

Supplementary Figure 1 Orbeye Olympus set-up.

Supplementary Figure 2 Video of PpIX agarose fluorescent gel in the half brain model.
