# Supplementary figures and images for: Automated online safety margin (GLIOVIS) for glioma surgery model
Source: Front Oncol. 2024 Apr 29;14:1361022. doi: 10.3389/fonc.2024.1361022 (PMC11089175; doi:10.3389/fonc.2024.1361022)

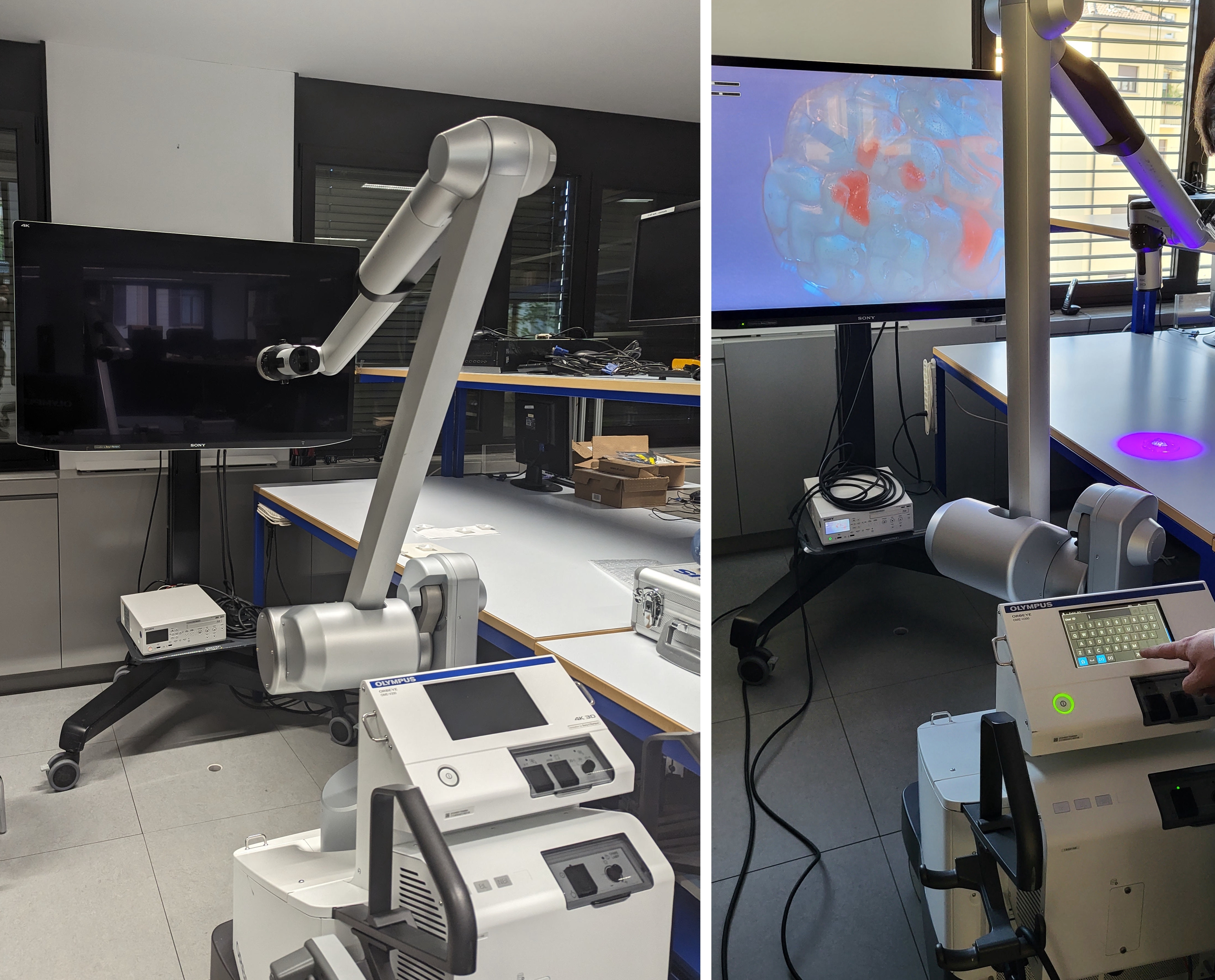

Supplement: Supplementary Figure 1 — Orbeye Olympus set-up. [file Image_1.jpg]
